# Autoantikörperdiagnostik bei idiopathisch inflammatorischen Myopathien

**DOI:** 10.1007/s00393-024-01476-0

**Published:** 2024-01-31

**Authors:** Robert Biesen, Udo Schneider, Antje Lindae, Rudolf Mierau

**Affiliations:** 1 Department of Rheumatology and Clinical Immunology, Charité – Universitätsmedizin Berlin, corporate member of Freie Universität Berlin and Humboldt-Universität zu Berlin, Charitéplatz 1, 10117 Berlin, Deutschland; 2 Institut für experimentelle Immunologie, affiliiert mit EUROIMMUN Medizinische Labordiagnostika AG, Lübeck, Deutschland; 3 Ehemals Labor an der Rheumaklinik Aachen, Aachen, Deutschland

**Keywords:** Myositis, Anti-Synthetase-Syndrome, Indikationen, Immunfluoreszenz, Etymologie, Myositis, Anti-synthetase syndromes, Indications, Immunofluorescence, Etymology

## Abstract

Idiopathische inflammatorische Myopathien (IIM) sind eine Gruppe seltener und heterogener Systemerkrankungen, die sich nicht nur an der Muskulatur, sondern auch an der Haut, den Gelenken und der Lunge manifestieren. Die Erstsymptome können isoliert und variabel auftreten, und so stellt die Diagnosefindung verschiedene Fachgruppen vor Herausforderungen. Da Autoantikörper mitunter die einzigen spezifischen Befunde sind, die zur Diagnose und einer angepassten Therapie führen, sind grundlegende Kenntnisse über diese unabdingbar. In dieser Arbeit erklären wir verfügbare Testsysteme, benennen die klinischen notwendigen Indikationen zur Veranlassung einer Autoantikörperdiagnostik, geben Informationen zu Etymologie, Antigenen, Synonymen und Erstbeschreibern, beschreiben die durch Myositisantikörper induzierte indirekte Immunfluoreszenz auf HEp-2-Zellen und geben die klinisch-serologischen Assoziationen wieder. Der Abgleich des Autoantikörperbefundes mit der klinischen Symptomatik und laborchemischen Befunden ermöglicht im Sinne einer Plausibilitätsprüfung, falsch positive oder auch falsch negative Laborbefunde zu identifizieren.

## Abstract (en)

Idiopathic inflammatory myopathy (IIM) is a group of rare and heterogeneous systemic diseases that manifest not only in the muscles but also in the skin, joints, and lungs. Initial symptoms can be isolated and variable and thus the diagnosis poses challenges to various specialist groups. As autoantibodies are sometimes the only specific findings that lead to the diagnosis and appropriate treatment, basic knowledge of them is essential. This article explains the available test systems, names the clinical indications necessary for the initiation of autoantibody diagnostics, provides information on the etymology, antigens, synonyms, and first descriptors, describes indirect immunofluorescence on HEp‑2 cells induced by myositis antibodies, and provides clinical-serological associations. The comparison of the autoantibody findings with the clinical symptoms and laboratory findings enables the identification of false positive or false negative laboratory findings in the sense of a plausibility check.

Idiopathische inflammatorische Myopathien (IIM) oder Myositiden sind seltene und heterogene Erkrankungen. Entgegen dieser historisch gewachsenen Begrifflichkeit manifestieren sich Myositiden nicht nur an der Muskulatur, sondern insbesondere auch an der Haut, den Gelenken und der Lunge. Es handelt also nicht um isolierte Muskelerkrankungen, sondern vielmehr um Systemerkrankungen. Die Beteiligung der Lunge bestimmt oft die Prognose. Die Inzidenz wird auf 5–10 pro 1 Mio. Einwohner pro Jahr geschätzt.

Die Diagnostik ist oft herausfordernd, da anfangs isoliert die Gelenke, die Haut, die Lungen oder die Muskulatur betroffen sein können und im diagnostischen Prozess neben der Rheumatologie auch Pneumologie, Neurologie, Dermatologie, Intensivmedizin, Radiologie, Pathologie und Allgemeinmedizin involviert sein können. Aus diesem Grund formieren sich regional interdisziplinäre sog. ILD (interstial lung disease) oder auch Myositis-Boards. Besonders tragisch sind schwere Verläufe mit plötzlich einsetzenden, isoliert auftretenden und schnell voranschreitenden interstitiellen Lungenerkrankungen, die zu respiratorischem Versagen führen und unerkannt letal enden können.

Die Einteilung der IIM ist aktuell Gegenstand intensiver Diskussionen. Anhand klinischer, laborchemischer und histologischer Merkmale unterteilt die deutsche S2k-Leitlinie [1] 5 Untergruppen: Anti-Synthetase-Syndrome (ASyS), Dermatomyositis (DM), Einschlusskörpermyositis (IBM), immunvermittelte nekrotisierende Myositis (IMNM) und Overlap-Myositis (OM). Bei dieser Einteilung spielt zunehmend auch der Nachweis charakteristischer Autoantikörper eine Rolle. Allerdings findet man nicht in jedem Fall einen dieser Myositisantikörper, sodass das Fehlen eines Autoantikörpernachweises die Diagnose einer IIM nicht ausschließt. Die klinische Definition Polymyositis ist zunehmend veraltet und wird heutzutage durch die eben genannten Entitäten abgelöst. Overlap-Myositiden treten bei anderen systemischen autoimmun-rheumatischen Erkrankungen wie z. B. der systemischen Sklerose auf. Bei den Dermatomyositiden werden häufig zusätzliche Attribute verwendet, die mit Autoantikörpern assoziiert sind. Das Auftreten einer DM vor dem 16. Lebensjahr – häufig, aber nicht ausschließlich, assoziiert mit Anti-NXP2-Antikörpern – definiert die juvenile DM (jDM). Tritt eine Neoplasie vor, während oder nach der Manifestation einer DM auf und lassen sich Antikörper gegen TIF-1γ, SAE1 oder NXP2 nachweisen, so besteht eine „cancer-associated dermatomyositis“ (CADM). Anti-Mi‑2- oder Anti-SAE1-Antikörper definieren die „klassische“ DM (cDM). Patienten mit Anti-MDA5-Antikörpern haben oft schwere Haut- und Lungenbeteiligungen, aber keine oder eine nur milde Muskelbeteiligung, sodass sich hier der Begriff amyopathische Dermatomyositis (aDM) etabliert hat.

## Labortechniken zum Nachweis von Myositisantikörpern

Im Folgenden sollen abschnittsweise 5 Labortechniken zur Detektion von Myositisantikörpern vorgestellt werden, wobei die letzten beiden nur kurz erwähnt werden sollen, da sie zwar wichtig, aber aktuell in der klinischen Praxis nicht oder kaum verfügbar sind.

*Die indirekte Immunfluoreszenz* auf HEp-2-Zellausstrichen (HEp-2-IFT, veraltet ANA-Test) ist ein einfacher und immer zu empfehlender Suchtest, dessen Befundung und Interpretation jedoch oft schwierig sind. Die Gründe dafür reichen von mittels HEp-2-IFT nicht nachweisbaren Antikörpern (MDA5, HMGCR, Ro-52 oder cN-1A) über schwache, teilweise fehlende oder variabel auftretende, fein gesprenkelte Muster im Zytoplasma, welche bei einer Routine-Level-Befundung mitunter nicht angegeben werden, bis hin zu Musterüberlagerungen bei Koexistenz anderer Autoantikörper, die die Befundung deutlich erschweren. Die durch Myositisantikörper verursachten Immunfluoreszenzmuster werden im späteren Abschnitt „Indirekte Immunfluoreszenz von Myositisantikörpern auf HEp-2-Zellen“ beschrieben.

*Immunblots* sind die für die Praxis relevanteste Nachweismöglichkeit von Myositisantikörpern und liefern in der Regel semiquantitative Messergebnisse. Gereinigte Antigene werden einzeln als Punkte oder Linien auf Membranen geblottet, sodass entsprechende Antikörper in einer einzigen Inkubation parallel nachgewiesen und identifiziert werden können. Da für Myositiden eine Vielzahl verschiedener Antikörper beschrieben ist, die in der Regel isoliert auftreten, ermöglicht diese Vorgehensweise eine umfassende Serologie mit guter Sensitivität. Auch wenn Myositis-spezifische Immunblots seit 2009 in Deutschland verfügbar sind, sind diese längst nicht überall zugänglich und zu oft universitären oder spezialisierten Zentren vorbehalten. In den letzten Jahren gab es mehrere Weiterentwicklungen dieser Myositisblots, die der zunehmenden Anzahl bekannt werdender Myositisantikörper Rechnung tragen. Falsch positive Befunde, insbesondere bei geringer Prätestwahrscheinlichkeit, und falsch negative Befunde können auftreten. Schwach positive Befunde, z. B. bei PM-Scl75, sind besonders kritisch zu prüfen [2]. Die sehr gute Kenntnis der verschiedenen Myositiskrankheitsbilder (s. auch „Klinische Assoziationen von Myositisantikörpern“) sowie mitunter der Abgleich mit dem HEp-2-Zell-Muster (s. auch „Indirekte Immunfluoreszenz von Myositisantikörpern auf HEp-2-Zellen“) erlauben die Plausibilitätsprüfung mit dem Ergebnis des Myositisblots, wodurch diese falsch positiven und falsch negativen Befunde identifiziert werden können. Nur ca. 70 % der IIM-Patienten weisen Myositisantikörper auf [2, 3], sodass eine Erweiterung des diagnostischen Spektrums der Blots wahrscheinlich ist.

*ELISA* werden nur selten benutzt und stehen aktuell nur zur Bestimmung von Antikörpern gegen Jo‑1, PM-Scl, U1-RNP, HMGCR und cN-1A zur Verfügung. ELISA-Testsysteme bieten die Möglichkeit der Quantifizierung. Dies kann insbesondere bei Anti-Jo‑1 und Anti-HMGCR für Verlaufsmessungen sinnvoll sein, da Korrelationen mit der Krankheitsaktivität belegt sind. Eine weitere Anwendungsmöglichkeit ist die Überprüfung eines unstimmigen Befundes im Myositisblot.

*Die Immunpräzipitation* gilt als Goldstandard zum Nachweis von Myositisantikörpern. Sie ist jedoch technisch schwierig, zeitaufwendig und verwendet radioaktive Reagenzien. Daher findet sie nur in wenigen Forschungszentren, wie z. B. in Bath (UK), Mailand (Italien), Stockholm (Schweden) und Baltimore (USA), Anwendung.

*Die Partikel-basierte Multi-Analytik-Technologie (PMAT)* verwendet farbcodierte paramagnetische Kügelchen, an welche die entsprechenden Antigene gekoppelt sind [4]. Sie benutzt Fluoreszenz als Antikörpernachweismethode und soll eine hohe Sensitivität und Spezifität gewährleisten. Diese Hochdurchsatz-fähige Technik liefert quantitative Ergebnisse und kann wie die Immunblots mehrere Antigene parallel erfassen. Ein CE-zertifiziertes System für die Myositisdiagnostik ist bisher nicht verfügbar.

## Indikationen zur Bestimmung von Myositisantikörpern

**Figure 1:**
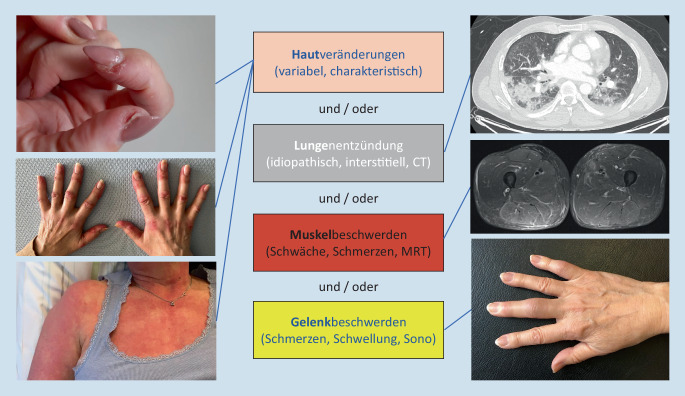

Die IIM sind eine Gruppe von Systemerkrankungen, die durch das Auftreten von variablen Hautveränderungen, einer entzündlichen Gelenkbeteiligung, einer Myositis oder einer interstitiellen Lungenerkrankung charakterisiert sind. Jede einzelne dieser Manifestationen (s. Abb. 1) bedarf auch bei isoliertem Auftreten und bei Fehlen einer gesicherten Diagnose einer Bestimmung von Myositisantikörpern. Eine parallele Bestimmung eines Myositispanels (via Blot oder PMAT) und einer HEp-2-IFT ist dann indiziert. Wichtig ist bei klinisch möglicher nekrotisierender Myositis, immer auch HMGCR-ELISA anzufordern, da Antikörper gegen HMGCR einerseits in der HEp-2-IFT typischerweise nicht sichtbar werden und andererseits auf zahlreichen Immunblots SRP und HMGCR bisher nicht enthalten sind, sodass hier hochspezifische Tests, die zur Diagnosesicherung führen, leicht verpasst werden können.

Selbstverständlich ist, dass primär auch alle anderen und oft häufigeren Differenzialdiagnosen für die vorliegende Symptomatik adressiert werden. Auch sei an dieser Stelle darauf hingewiesen, dass die Autoantikörperbestimmung nur ein „Diagnostikbaustein“ ist und in der Abklärung einer IIM je nach Manifestation eine Biopsie von Haut, Muskulatur oder Lunge und eine Bildgebung mittels MRT der Muskulatur oder CT der Lunge diagnostisch wertvolle Informationen liefern können.

Aufgrund fehlender Sensitivität und Spezifität haben CRP und BSG keinen diagnostischen Stellenwert bei der Abklärung einer fraglichen IIM.

Isolierte laborchemische Erhöhungen der Kreatinkinase (CK) ohne Vorliegen einer klinischen Symptomatik sollten nur dann durch eine Antikörperdiagnostik adressiert werden, wenn andere häufigere Ursachen wie z. B. Sport, Statinexposition, Schilddrüsenerkrankung, Anfallsleiden, Makro-CK, hohe Muskelmasse oder ein Trauma ausgeschlossen wurden und die CK-Erhöhung über das 3fache der Norm über 4 Wochen besteht. Neben dem Veranlassen eines kostenintensiven Myositispanels besteht das Risiko, im Falle eines falsch positiven Befundes in Erklärungszwang zu geraten. Falsch positive Befunde werden in erster Linie nicht durch Testsysteme, sondern durch ungerechtfertigte Diagnostikanforderungen verursacht.

## Erstbeschreibung und Namensgebung von Myositisantikörpern

Bei der Mehrzahl der Patienten mit IIM können mittlerweile charakteristische Autoantikörper nachgewiesen und den oben erwähnten IIM-Gruppen zugeordnet werden. Die Autoantikörper sind somit hilfreich für die Diagnosefindung und für die Zuordnung zu klinisch und prognostisch abgrenzbaren IIM-Subsets. Traditionell werden diese Antikörper in „Myositis-spezifische“ (MSA) und „Myositis-assoziierte“ (MAA) aufgeteilt. Letztere wie Anti-Ku-, -U1-RNP oder -PM-Scl treten v. a. bei Vorliegen anderer Kollagenosen auf. Diese Abgrenzung ist historisch gewachsen und erscheint nur bedingt gerechtfertigt: Auch „MSA“ wie die Antisynthetasen oder Anti-MDA5 gehen nicht immer mit einer Myositis als Symptom einher, und es gibt auch Patienten mit „MSA“, die ähnlich wie diejenigen mit „MAA“ die Klassifikationskriterien anderer Kollagenosen wie der systemischen Sklerose erfüllen.

**Figure 2:**
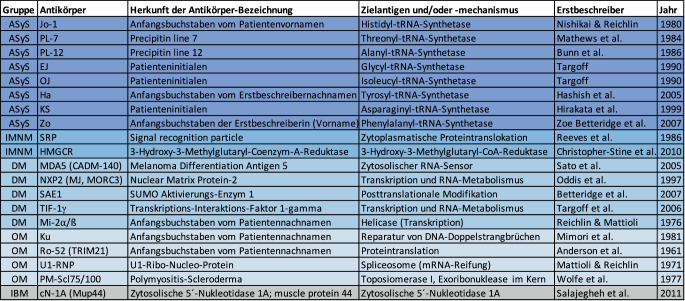

Die Myositisantikörper sind in Abb. 2 unter den Myositisgruppen ASyS, NM, DM, OM und IBM aufgeführt. Anti-Jo1- und -Mi-2-Antikörper waren die ersten beschriebenen MSA und wurden von der Arbeitsgruppe um Morris Reichlin beschrieben [5, 6]. Sieben von 16 MSA (44 %) wurden immerhin in den letzten 18 Jahren beschrieben.

Die Groß- und Kleinschreibung der Antikörper ist wichtig und liefert Hinweise zur Herkunft. So sind Ro, Mi und Ku jeweils die Anfangsbuchstaben von Patientennachnamen. Interessanterweise leiten sich Anti-Ha-Antikörper vom Nachnamen Hashish des Erstbeschreibers und Anti-Zo-Antikörper vom Vornamen Zoe Betteridge, ebenfalls Erstbeschreiberin, ab [7]. Zwei einzelne Großbuchstaben entsprechen immer den Initialen des Indexpatienten (EJ, OJ, KS). Die übrigen Bezeichnungen entsprechen Abkürzungen des Antigens (z. B. SRP oder c1-NA), des klinischen Phänotyps (PM-Scl) oder der Technik (PL‑7, PL-12), in der sie erstmals identifiziert wurden (vgl. Abb. 2).

## Indirekte Immunfluoreszenz von Myositisantikörpern auf HEp-2-Zellen

Im Gegensatz zum systemischen Lupus erythematodes schließt das negative Ergebnis eines HEp-2-IFT eine IIM nicht aus. Der Test hat jedoch seinen Stellenwert als Suchtest und zur Plausibilitätskontrolle.

Es ist zum Verständnis hilfreich zu erwähnen, dass die als Substrat verwendeten HEp-2-Zellen Epithelzellen einer Larynxkarzinomzelllinie sind, welche aufgrund ihres großen Zellkernes primär für die Detektion von antinukleären und *nicht* von antizytoplasmatischen Antikörpern ausgewählt wurden. So werden einige der Zielantigene in diesen Zellen gar nicht oder nur sehr schwach exprimiert (z. B. cN-1A, HMGCR, MDA5), und Antikörper gegen diese Antigene sind damit nicht nachweisbar. Im Gegensatz dazu lassen sich beispielsweise Antikörper gegen HMGCR mit Leberzellen als Substrat mit der Immunfluoreszenz problemlos darstellen.

**Figure 3:**
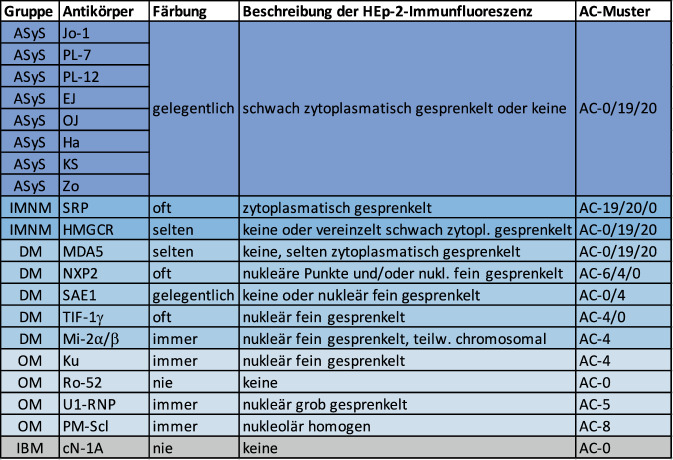

In Abb. 3 werden die Myositisantikörper den entsprechenden Immunfluoreszenzmustern auf HEp2-Zellen und der Nomenklatur nach internationalem Konsens für antinukleäre Antikörper (ICAP) gegenübergestellt [8]. Antikörper gegen Mi2, Ku, U1-RNP und PM-Scl induzieren immer eine HEp-2-Immunfluoreszenz. Daraus ergeben sich 2 diagnostisch wertvolle Schlussfolgerungen:

1. Nur die Mi-2-positive DM und Ku/U1-RNP/PM-Scl-positive OM können mit einem negativen HEp-2-IFT ausgeschlossen werden.
2. Positive Befunde in einem Myositisblot für Mi2, Ku, U1-RNP und PM-Scl ohne korrespondierende Immunfluoreszenz/AC-Muster sind anzuzweifeln.

Die Existenz von allen anderen Myositisantikörpern lässt sich durch einen negativen HEp-2-IFT nicht ausschließen. Daraus ergibt sich, dass potenzielle falsch positive Befunde eines Myositispanels nicht in einer laborchemischen Plausibilitätskontrolle mittels HEp-2-IFT zu identifizieren sind. Der Vollständigkeit halber soll nicht unerwähnt bleiben, dass im Linienblot positive Antikörperbefunde, für die kommerzielle ELISA verfügbar sind (Jo‑1, HMGCR, PM-Scl, U1-RNP, cN-1A), mit diesen überprüft werden könnten.

## Klinische und laborchemische Assoziationen von Myositisantikörpern

Durch den Nachweis eines Myositisautoantikörpers lassen sich IIM-Patienten klinisch homogeneren Subgruppen zuordnen, die sich hinsichtlich Schweregrad, Organbeteiligungen, Epidemiologie, Prognose und Therapieansprechen voneinander unterscheiden.

Kenntnis dieser klinisch-serologischen Beziehungen kann – v. a. bei oligosymptomatisch beginnenden Fällen – den Fokus auf zu suchende oder im Verlauf zu erwartende Symptome oder Organbeteiligungen lenken und häufig auch bei der Abschätzung der Prognose helfen.

**Figure 4:**
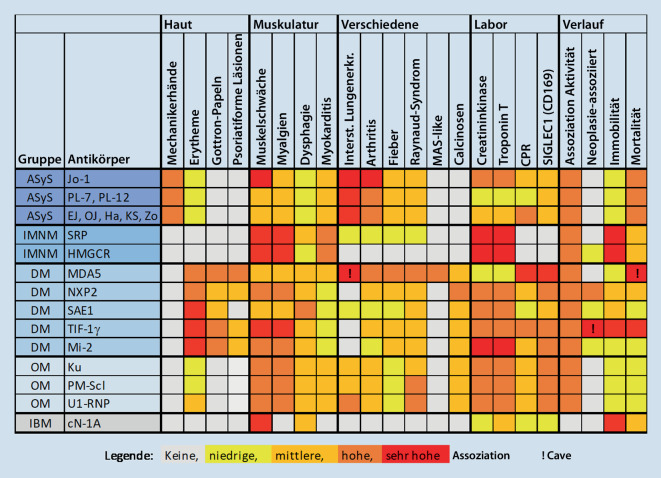

Darüber hinaus bietet die Kenntnis der durch Autoantikörper definierten IIM-Subsets auch die Möglichkeit, die Plausibilität eines Autoantikörperbefundes zu kontrollieren, zumal die Mehrzahl der positiven Befunde in einem Myositispanel nicht durch ein anderes Testsystem validiert werden kann. Damit dies möglich wird oder auch um vorab durch Erfassung des Krankheitsmusters eine konkrete klinische Verdachtsdiagnose zu generieren, um welche IIM oder Myositisgruppe es sich handeln könnte, sind in Abb. 4 grundlegende klinische und laborchemische Assoziationen zu den jeweiligen Myositisantikörpern dargestellt. Neben Standardlaborwerten wie CK, Troponin T und CRP haben wir auch SIGLEC1 als Biomarker für Typ-I-Interferon ergänzt [9]. Abb. 4 veranschaulicht zudem die Heterogenität und den Systemcharakter von idiopathisch inflammatorischen Myopathien.

Da es sich hierbei jedoch um ein weites und sich schnell entwickelndes Feld handelt, in dem Erkenntnisse von seltenen Krankheitsbildern oft nur über Multicenteranalysen gewonnen werden können, liegt es in der Natur der Sache, dass Abb. 4 unvollständig ist und Ungenauigkeiten oder Fehler enthalten sein können, obwohl die Autoren diese Tabelle sorgfältig recherchiert und mit persönlicher Erfahrung abgeglichen haben. Da uns zudem keine relevanten klinischen Unterschiede zwischen Mi-2α und Mi-2β, PL‑7 und PL-12 und den sehr seltenen EJ, OJ, Ha, KS und Zo bekannt sind, haben wir diese Antikörper zwecks Übersichtlichkeit zu Gruppen zusammengefasst.

## Zusätzliche IIM-modifizierende Autoantikörper

Mit wenigen Ausnahmen zeichnen sich IIM-Patienten durch den Nachweis nur eines der in Abb. 4 gelisteten Antikörper aus. Relativ häufig finden sich jedoch zusätzlich zu den bisher genannten Myositisantikörpern weitere IIM-unspezifische Autoantikörper, die die klinischen Assoziationen modifizieren können.

Dies gilt insbesondere für Antikörper gegen Ro-52 (Synonym TRIM21). Sie wurden zusammen mit Anti-Ro-60 ursprünglich im Zusammenhang mit dem Sjögren-Syndrom und dem SLE beschrieben. Ro-52 und Ro-60, benannt nach den ersten beiden Buchstaben des Nachnamens der Indexpatientin und der Molmasse, sind biochemisch, funktionell und hinsichtlich der Lokalisation im HEp-2-IFT komplett verschieden. Anti-Ro-52 sind – selbst bei Gesunden – ziemlich häufig auffindbar und damit in keiner Weise spezifisch für IIM. Ihr alleiniger Nachweis trägt somit nicht zur Diagnosefindung einer IIM bei. Der parallele Anti-Ro-52-Nachweis zusätzlich zu einem anderen Myositisantikörper hingegen ist von großer Bedeutung. So finden sich Anti-Ro-52 bei ca. einem Drittel der IIM-Patienten. Die Prävalenz ist bei durch ASyS-Ak charakterisierten Patienten mit 40–70 % noch höher. Vielfach wurde berichtet, dass Anti-Ro-52-Ak dabei mit häufigerer und/oder rascher progredienter ILD, häufigeren Rezidiven und/oder höherer Mortalität assoziiert sind [10]. Somit zeigt der Nachweis von Anti-Ro-52 zusätzlich zu einem IIM-Antikörper eine generell schwerere (Lungen‑)Erkrankung an.

Antikörper gegen citrullinierte Peptide/Proteine (ACPA) sind die wichtigsten Marker-Ak der rheumatoiden Arthritis (RA); seltener kommen sie auch bei anderen entzündlich rheumatischen Systemerkrankungen vor und sind bei diesen oft mit Arthritiden assoziiert. Bei ASyS-Patienten wurden Zusammenhänge von ACPA mit aggressiverer, früher auftretender und/oder erosiver Arthritis oder einer RA-typischen Gelenkverteilung ermittelt [11]. Ein monosymptomatisch mit Arthritis beginnendes ASyS kann einer RA ähneln und aufgrund eines ACPA-Nachweises als seropositive RA gedeutet werden. In solchen Fällen weist der Nachweis von Anti-Jo‑1 oder anderen Antisynthetasen auf das hohe Risiko des Auftretens weiterer ASyS-Symptome wie Myositis und Lungenfibrose im Verlauf hin.
