# Erratum zu: Autoantikörperdiagnostik bei idiopathisch inflammatorischen Myopathien

**DOI:** 10.1007/s00393-024-01492-0

**Published:** 2024-02-21

**Authors:** Robert Biesen, Udo Schneider, Antje Lindae, Rudolf Mierau

**Affiliations:** 1 Department of Rheumatology and Clinical Immunology, Charité – Universitätsmedizin Berlin, corporate member of Freie Universität Berlin and Humboldt-Universität zu Berlin, Charitéplatz 1, 10117 Berlin, Deutschland; 2 Institut für experimentelle Immunologie, affiliiert mit EUROIMMUN Medizinische Labordiagnostika AG, Lübeck, Deutschland; 3 Ehemals Labor an der Rheumaklinik Aachen, Aachen, Deutschland

**Erratum zu:**

**Z Rheumatol 2024**

10.1007/s00393-024-01476-0

In diesem Artikel waren die Adressdaten und der Interessenkonflikt für die Autorin Antje Lindae falsch angegeben. Korrekt ist:

Affiliation: Institut für experimentelle Immunologie, affiliiert mit EUROIMMUN Medizinische Labordiagnostika AG, Lübeck, Deutschland.

Interessenkonflikt

A. Lindae ist bei EUROIMMUN, einem Unternehmen, das diagnostische Tests und Geräte herstellt, angestellt. A. Lindae hat in Zusammenhang mit dem vorliegenden Beitrag keinen potenziellen oder tatsächlichen finanziellen Gewinn erzielt.

Der Originalbeitrag wurde korrigiert.

